# What do we transfer in case discussions? The hidden curriculum in medicine…

**DOI:** 10.1007/s40037-013-0101-0

**Published:** 2013-12-24

**Authors:** Yolande Witman

**Affiliations:** Postgraduate School in Organization Studies and Change Management, Sioo, Utrecht, the Netherlands

**Keywords:** Hidden curriculum, Medical habitus, Teaching rounds, Power relations, Reflective practice

## Abstract

Medical students and junior doctors learn according to the formal curriculum, but they also learn unwritten rules, the specific logic of the medical world, through a socialization process called ‘the hidden curriculum’. The result of the process seems to be an exclusive professional identity, the medical habitus. This article underlines the importance of the hidden curriculum in medicine, especially in meetings where patients are discussed. These case discussions, common daily rituals in medical practice, demonstrate the dynamic interaction of learning and practice within the medical world. The article illustrates how medical core values are transferred informally and implicitly, and the role of power relations in this process. Not only are residents being assessed and trained in case discussions, but also staff are assessing each other and learning continuously. Therefore, these meetings might significantly contribute to self-regulation in medicine. The significance of the hidden curriculum should not be underestimated. Insights into the dynamics of case discussions may help medical specialists to make the most of this moment of learning and to avoid the pitfalls, for the benefit of both residents and experienced medical specialists.

## Introduction

The modernizing of professional education and training in many fields is characterized by formalizing educational goals and making professional values more explicit, for instance through the introduction of competence management and portfolios. This also applies to medical training. Medical students and junior doctors learn according to the formal curriculum. This is defined by goals, compulsory subject matter for examinations, competences and so forth. But they also learn the unwritten rules, the specific logic of the medical world. They learn to behave according to the specific professional and collegial norms of the profession, which is ‘as important as the science and art of medicine, although it is learned through a socialization process rather than classroom lectures’ [1]. This intense socialization process is also called ‘the hidden curriculum’ [2–8]. It works through individualization, but also through separation from the ‘normal’ world [9–11].The hidden curriculum starts in the first year of medical training and intensifies when interns and residents are participating in patient care together with medical specialists: clinical contact is an important part of the process of socialization, as it allows students to enter the community of practice of the medical profession [4, 12]. The result of this process might be conceptualized as a common professional identity, the medical habitus.

We used the concept of habitus in our research of medical leadership in the hospital organization [8]. The concept of habitus comes from the range of thought of Bourdieu [13] and can be considered the internal model of social reality in an individual. The habitus develops in a socialization process and can be defined as a system of dispositions: durable, subconscious schemes of perception and appreciation that activate and point the way to practice. Dispositions are not directly visible, but they reveal themselves in certain visible patterns of behaviour, manners and beliefs: in practices. The habitus ensures that people take their social world for granted. Elias speaks of a ‘second nature’ [14]. Indeed, the socialization process in which the habitus comes into being is therefore to be considered a form of social control: during the hidden curriculum, external rules become internalized and also the power relations are transferred.

Various studies of this socialization process in the medical world, which are set in different times and places, show striking similarities that point to a more or less exclusive professional identity, the medical habitus [2, 8–11, 15–19].

The medical habitus, the vital way of thinking and acting, is usually transferred informally and implicitly in practice. The impact of this way of learning is therefore underrated, as a result of which changes in curricula have insufficient or even harmful effects.

This paper will focus on the hidden curriculum; it describes what medical students and junior doctors learn and how they develop ‘a feel for the game’ in meetings where patients are discussed. These discussions, common daily rituals in medical practice, demonstrate the mostly implicit, informal patterns and power relations in the medical world. As interns and residents participate in this meeting together with the medical specialists, patient care and medical training are inextricably bound to each other. Therefore the case discussion is an excellent setting to illustrate the hidden curriculum with the implicit patterns, power relations included.

## Case discussions

In case discussions, doctors discuss patients they have seen for a consultation, patients they hospitalize or operate on, or patients who have had complications. These patients are not present in person; stories and information of the patients are discussed. Meetings with these discussions take place regularly; they can change in frequency, composition, style and atmosphere. Their length depends on the available time (from 15 min to 2 h), the objective of the meeting and the size of the department: teaching rounds, morning report, morbidity and mortality review, multi-disciplinary team meeting (MDT). Time is limited, for instance, in a meeting on Monday morning when many patients have to be discussed before clinics, patient rounds and operations start.

The case discussion shows characteristic moments that I discuss chronologically; however, in practice the different phases can be merged. The presentation of a patient is always the starting point, followed by questioning, discussion and decision-making.

## The presentation of the patient, the story of the doctor

Usually residents present the story of the patient they have seen for a consultation in the (outpatient) clinic or for an admission. The presentation of the patient to colleagues who have not seen the patient in person has a regular structure. The resident describes the patient’s main symptom, the results of the anamnesis, physical examination, differential diagnosis, additional examinations, diagnosis and a proposal of treatment.

The case presentation can be considered the fundamental medium of clinical thought and discourse [20]. In this story, the doctor ‘translates’ the patient’s symptoms and the course of the disease in the medical line of reasoning [21]. In their presentation the residents show the extent to which they have mastered this translation. Their colleagues see whether the residents have a firm grasp of the medical business.

How can mastery of the medical business be shown? Doctors find this difficult to explain. There are different aspects. The content of the story is of importance, but also the form and the cadence: the way in which the story is told. It is important to separate essentials from side issues, to use neither too many nor too few words and not to mention unnecessary details.^1^ The story must have a proper structure and medical terms should be pronounced correctly.

Also the attitude is important. The presentation should not be too unsure, nor too arrogant; the tone has to fit the phase of training and experience. Residents are expected to show progress during their residencies. At a certain moment they have to present from memory, by which they show they have mastered the medical knowledge.

The presentation does not only tell the story of the patient, it is also the story of the doctor: the doctor presents *his* story of the patient and by that he presents himself. He shows his ability to translate the story of the patient and the results of the examinations, his ability to use clinical reasoning to make a proper diagnosis and to choose the right treatment. He shows to what extent he has mastered the *clinical disposition*: to see persons as patients. Whereas a non-doctor may see a woman with remarkable striking eyes, a doctor will see someone with the symptoms of a disease of the thyroid gland: the doctor sees a patient. Like a detective, the doctor looks for complaints and symptoms that point the way to a disease that has to be cured. Colleagues may hear if there are any inconsistencies in the story about the patient, omissions or inaccuracies. This leads to the next characteristic phase of the case discussion: asking a lot of questions.

## Questions, questions, questions

After the presentation the responses of the chair and the other specialists interestingly often take the form of questions. These questions have different purposes: obtaining more information, testing, criticizing and showing off one’s knowledge.

If the inquirer wants to test someone’s knowledge, he asks a question he already knows the answer to: a specialist asks the resident to describe the findings on an X-ray or the criteria of a particular operation. He may also ask the resident about additional examinations and considerations for diagnosis or treatment. For example: ‘*What was the indication for the ultrasound scan?*’^2^ The inquirer is not so much interested in the exact answer, he wants to know if the habit of thought is logical. These questions are especially aimed at clinical reasoning.

When a resident forgets to mention an element in his presentation, he is pointed to this omission with a question: ‘*Was there red blood loss per anum?*’ *Is there a positive family anamnesis? Are there no signs of portal hypertension?*’

The element that the resident did not mention may be an absent positive finding, but also a present negative finding. The inquirer checks if the resident has asked himself these questions and underlines their significance. The answers to these questions are necessary for the right steps in the thought process and for the differential diagnosis.

The question aimed at testing may switch to a form of criticism. The question: ‘*Did you consider performing a puncture?*’ may have a learning goal. This question gives the resident a suggestion. But it may also mean that he should have considered it, or that he should have expressed his considerations with regard to the puncture.

Through critical questions staff also make clear that the resident did not distinguish essentials from side issues: ‘*In one sentence please?*’

The critical question also happens to be an important collegial manner between staff members: criticism is generally disguised by questions. It does not mean that physicians do not criticize each other, but they do not criticize each other openly. The critical questions function well for the good listener. Questions prevent a colleague from losing face, a very threatening situation for a physician. This collegial manner refers to the *collegial disposition*.

Asking the right questions commands at least as much respect as being able to give the proper answers. This is a way to demonstrate competence. A good question may show that the storyteller overlooked something that the inquirer came to think about. A question may also be the introduction to the story of an interesting patient whom the inquirer himself has once treated, or to bring up a study or guidelines. Regarding this last aspect the *scientific disposition* is revealed: to see medicine as a scientifically founded discipline.

In the next phase doctors put forward arguments in the discussion.

## Discussion: good arguments with respect to content

When doctors disagree about a diagnosis or treatment, fierce discussions may arise. Questions and arguments are brought up with ardour. They seem to spring up when talking about a complicated patient: passionately and enthusiastically the Sherlock Holmeses^3^ are busy trying to catch the offender in the act. Just as it is a great challenge for the detective to find out who committed the crime, so it is for the doctor to find out which disease may explain the symptoms with enough certainty that the patient will be cured. How could the symptoms, an inexplicable course of a disease or the failure of a treatment be explained? These are all puzzles that have to be solved.

While putting forward arguments, one draws from experience and scientific knowledge.

An argument from personal clinical experience can start as follows: ‘*I just saw someone…*’ Hereafter follows a plea about the matter which does or does not support the diagnosis or treatment in the present case.

Other arguments refer to the state of affairs in science. One calls upon evidence-based medicine and the results that are translated to guidelines and protocols. This knowledge may be put forward, for example, to perform a particular procedure, to make one diagnosis more plausible than another, or to consider a special treatment more sensible than another. The different arguments from experience and scientific knowledge can sometimes conflict.

Guidelines give residents something to hold on to, while members of the staff who by definition have more experience, sometimes take a disparaging view of guidelines. They put the value of these guidelines in perspective or they point to their negative effect. But arguments based on experience or knowledge may also go together or even reinforce each other.

Another argument that emerges is the experienced urgency to have to do something for the patient. If a doctor does not expect surgery to be of much help to his patient, he may still decide to perform a risky operation, because he feels the urgency to do something for his patient. Doing nothing feels like a failure, as if they did not do the best they could. Arguments about time, effort or costs are not the point; the real question is whether a treatment is clinically relevant and whether a patient will benefit.

From this we derive *the professional disposition*: to see oneself as personally responsible.

## Making decisions on behalf of one’s own patients

Often the decision process passes off implicitly. When there are no obvious irregularities, the next patient will be presented without much comment, sometimes only: ‘*Okay, next.*’ Apparently, the decisions about diagnosis or treatment are adopted tacitly. The moment of the decision may be unclear. After the last arguments there is no reply, so this happens to be the conclusion, or perhaps the most relevant advice. The chair of the meeting or the patient’s ‘own’ doctor ends the discussion with: ‘*Okay, this is what we*’*ll do,*’ or: ‘*Okay, this fits the picture most closely.*’

The position of the patient’s own doctor becomes clearly noticeable when something is not clear or with criticism: ‘*Whose patient is this?*’ or: ‘*Who knows this patient?*’ Or the resident who defends himself in advance when he has to present the patient of a colleague: ‘*I will read from another man*’*s work.*’

The patient’s own doctor ‘knows’ his patient, may pass judgement and may take a decision. Deciding with regard to one’s own patient reveals another important collegial manner, referring to the *collegial disposition*: not to give orders. Whoever decides that an operation is suitable, the responsibility is in the hands of the operating doctor and when something goes wrong, he or she is to blame. These manners are related to the *professional disposition* in accordance with physicians who decide for themselves—give themselves orders—and are supposed to take responsibility. This claim to make one’s own decisions refers to the importance of autonomy.

The patient’s own doctor decides, but the opinions of colleagues may actually influence the decision: the decision-making during the case discussion is a social process. Although a manager in an interview sighed, finding it incomprehensible that doctors hardly assess each other, these case discussions are to be considered one great assessment. Participants constantly assess themselves, their colleagues and the policy of the group as a whole through questions and the exchange of arguments. They may learn from mistakes and adjust bad medical management. They use one another to get more certainty and to rank with each other. They try to reach consensus, they share the responsibility for the decision, as it were. This collegial manner refers to another unwritten rule of the *collegial disposition*: to be collegial and to be loyal to each other. It is important to cover for colleagues in different circumstances:

A specialist

If you have a problem, you should immediately have someone to fall back on, otherwise patients are in danger. Collegiality is the key in safety issues. It means that you back each other up, that you don’t let each other down. It is teamwork.

‘To be collegial’ includes covering for colleagues in case of illness, conference attendance or private obligations, often without clear agreements about what will be done in return. To be loyal means, for example, not to criticize each other outside the group. The principle of reciprocity is important here. Although the strategies of the collegial disposition are clearly important, this does not mean that they are always followed. This leads to a delicate balance between cooperation and competition [8].

## The medical habitus

From the characteristic patterns of behaviour in the case discussion, four dispositions of the medical habitus can be distilled: the clinical, the scientific, the professional and the collegial disposition (Table 1). Not only the habitus, but also the power relations are transferred during the case discussions.

**Table 1:** The medical habitus

Through the *clinical* disposition physicians perceive a person as a patient. To acquire, preserve and develop this disposition, the doctor has to see many patients and acquire experience, because ‘every patient is different’. The disposition manifests itself in specific patterns of reasoning during the presentation of the patient in case discussions, in asking specific kinds of questions and in certain arguments during discussions
The *scientific* disposition means that physicians see medicine as science. Physicians generate and apply scientific knowledge by ‘seeing’ their patients. This disposition reveals itself in book learning and in speaking the scientific medical jargon in the early years of training, and later on in the use of scientific knowledge in patient care, in reading scientific literature, and in visiting research meetings and conferences
The *professional* disposition means that physicians perceive themselves as personally responsible for their patients. Physicians put the interests of the patient first and claim the competence to make clinical decisions on behalf of their own patients. This disposition manifests itself in arguments regarding choices for treatment of patients, in working hard, sometimes in suffering physical deprivation, and in being able and having the courage to decide. The professional disposition is to be considered as the professional conscience of the physician
The *collegial* disposition refers to the inextricable relationship between group membership and individual performance. This disposition gives rise to three forms of collegial manners, mostly in accordance with unwritten rules. Firstly: to give no orders, not to control each other, decision-making by consensus. Secondly: to be collegial: to do each other favours and to be loyal to each other. Thirdly: not to criticize each other openly. Criticism is generally disguised in questions

## Power relations

During these meetings the hierarchy in the medical world becomes visible: the place and attitude of people reveal their position in the group. Staff take their place at the table, residents are lined up at the wall, decorated with paintings of famous predecessors.

The meaning of authority becomes even clearer when it is not respected: the resident who takes the ‘wrong chair—the one informally reserved for seniors—offends: he has no sense of proportion. This may seem a trivial matter for an outsider, but the resident does not only sit in the wrong place literally, but figuratively as well. The irritation lies in the lack of respect for seniority and experience, which is so highly valued in medicine. His lack of respect for seniority could also mean that the resident falls short in asking for supervision or advice.

But the hierarchy also becomes manifest indirectly. The residents present the patients and seniors ask the questions to test their knowledge, but not the other way round.

The questions may give the residents a hard time in two ways: they can fail because they may not know the answer to an important question. Or they do know the answer, but they do not mention it, because they have overlooked its importance. In the words of a resident: ‘*When I get a question that I can answer, I nevertheless think: shit, I made an important mistake.*’ He forgot to mention a relevant positive or negative result, by which he could show his competence in the right line of reasoning. Because of this he may feel that he is losing face and is ashamed.

Even though the critical question might be frightening, questions are also appreciated. The question and answer game between junior and senior should, in the end, take place in the head of the physicians when they sit in their consulting rooms, alone with their patients (see also Sinclair [10]). Questions function as instruments of testing knowledge and of transferring the art of clinical reasoning as well.

In the phases of discussion and decision-making senior specialists discuss complicated patients, while residents are silent listeners. But these residents do see how specialists sit up straight when they discuss a complex patient, who does most of the talking, which arguments are decisive and who determines the decision-making process. These power relations give rise to a clear hierarchy, not only between specialists and residents, but also among specialists. There is talk of A and B surgeons, of first- and second-rate specialists. The clinical disposition plays a leading role in this informal hierarchy and also in the leadership of department heads [8]. Clinical authority is connected to clinical experience and seniority: these physicians have seen a lot of patients in the course of their careers and are therefore able to diagnose, treat and operate on particular patients. A physician with authority is to be considered, speaking in Bourdieu’s terms, a wise man, i.e. someone whose authority is mostly founded on the medical habitus.

These wise men in the group can put their stamp on case discussions, but also during rounds or in the operating theatre. They actively ask questions and give advice, not only to the residents, but also to their staff. Staff ascribe authority to them.

## Teaching by humiliation?

Training in medicine is sometimes criticized due to reports of ‘teaching by humiliation’ [10]. Even when staff do not humiliate them, residents may fail in their own eyes. Feelings of shame and humiliation are violent emotions that usually have a negative connotation. Nevertheless, shame also has another function. It can be a signal that the bond with the group is threatened [23]. To avert this threat, shame may guide someone to display the desired behaviour. Elias speaks of the pain threshold that contributes to the adaptation of behaviour and finally also to that of the habitus [14]. Consequently, a physician is doing his level best to prevent losing face. The fear of shame functions as an important machine of social control and plays a part in the establishing of the medical habitus as the professional conscience.

## The hidden curriculum, an underrated phenomenon

In this paper I have answered the question: what and how do medical students and junior doctors learn in case discussions?

Even though the training of doctors is as formalized and explicit as possible, the medical habitus arises from participation in daily practices such as case discussions. In the hidden curriculum the medical habitus is transferred, as the foundation for clinical, scientific, collegial and professional behaviour, including power relations. That is more than just social and moral behaviour in the narrow sense! Referring to Bourdieu the hidden curriculum in medicine is to be considered ‘a slow process of co-option and initiation which is equivalent to a second birth’ (Bourdieu [13], p. 68).

The importance of learning through this hidden curriculum should not be underestimated, especially at a time when the notion of ‘competence-based learning’ also dominates the training of doctors.

Exclusive attention to a specific set of desired competences might obscure the issues that are not made explicit and leads the attention away from the hidden way of learning, where craftsmanship and social behaviour are not easily separated. Power relations might be neglected, while they are so important in practice and for learning. Moreover, competence-based learning is based on the assumption that formalizing educational goals and making more explicit formulations of professional values is always better than the hidden way of learning. This could neglect the way of learning that happens anyway in the day-to-day practice. It might explain why many formal educational reforms in the medical world gave few actual changes [24]. We^4^ developed a workshop to bridge the gap between the formal and the informal curriculum in case discussions, which may help teams of specialists and residents to explore the hidden curriculum in their own case discussions, and thus may function as a first step towards optimizing these meetings (Table 2).

**Table 2:** Workshop ‘case discussions and the hidden curriculum’

In the workshop we aim at raising the consciousness of the dynamics of case discussions. We discuss the phenomenon of the hidden curriculum, the different characteristic phases of the meeting, the role of power relations and the significance of a competent chair of the meeting. We practise with different interventions for chairs in a simulation of these meetings, as to teach medical specialists for instance: to structure the meeting; to support learning by asking questions; to make considerations aloud (in this way they make their tacit knowledge explicit); to use the different functions of questions; to manage ‘teaching by humiliation’. We stress the fact that all medical specialists need to realize that they function as role models all the time
A point of concern that is often mentioned is the time that is available for these meetings, especially in a time where non-paid activities such as mutual and multidisciplinary meetings are under pressure

In case discussions, knowledge production and reproduction occur [8, 15]. Case discussions are important to acquire experience and for continuous education. Many patients are discussed in a single meeting, and these patients, even though ‘on paper’, contribute to the amount of patients that one has seen: ‘*The mother of all learning is not repetition*—*it is variation. It is when ten or a hundred different lamps have been pointed out to him that a child learns the word and the concept of lamp.*’^5^

Story-telling may explore the practice-based tacit knowledge [25]. Doctors exchange practice stories and improve their practice continuously through reflective practice [26]. Reflection appears when ‘knowing-in-action—the knowledge by which someone practices—leads to an unexpected result. Two kinds of reflection may occur: ‘reflection-in-action’, to think about the adjustment of the practice and ‘reflection-on-action’: the reflection after the experience, which means that the practice becomes a learning experience. This reflection occurs in case discussions.

Not only are residents being assessed and trained, but also staff are assessing each other and learning continuously. Ericsson, who did extensive studies of ‘expert performance’ in different professions, underlines the significance of ‘deliberate practice’ to continue upgrading the level of performance [27]. Case discussions might be considered to be an example of deliberate practice. Therefore these discussions might form a significant contribution to self-regulation in medicine. As Kenny et al. [28, p. 1209] tells us: ‘*Excellence in professional practice is learned in and through experience and critical reflection on its expression in the clinical encounter*’.

To conclude, insights into the dynamics of the hidden curriculum may help medical specialists to make the most of this moment of learning in case discussions and avoid the pitfalls, for the benefit of both residents and experienced medical specialists.

## Essentials

- The importance of the hidden curriculum in medicine should not be underestimated
- The medical habitus arises from participation in daily practices such as case discussions
- Power relations in medicine are important in practice and for learning
- The case discussion is a learning experience for both residents and staff
- The case discussion contributes to the self-regulation in the medical world

## Footnotes

1 ‘Clinical reasoning, rather than factual regurgitation’ [22].

2 Quotations without reference come from ‘*De medicus maatgevend. Over leiderschap en habitus*’ (Witman [6]).

3 Sir Conan Doyle, the author of Sherlock Holmes, was also a physician.

4 In cooperation with the special interest group for postgraduate medical education of the NVMO.

5 Oratie Bodil JoÅNnsson, Certec, Department of Design Sciences, Oct 8, 1999.
